# Modeling Exon Expression Using Histone Modifications

**DOI:** 10.1371/journal.pone.0067448

**Published:** 2013-06-25

**Authors:** Shijia Zhu, Guohua Wang, Bo Liu, Yadong Wang

**Affiliations:** Center for Biomedical Informatics, School of Computer Science and Technology, Harbin Institute of Technology, Harbin, China; Ludwig-Maximilians-Universität München, Germany

## Abstract

Histones undergo numerous covalent modifications that play important roles in regulating gene expression. Previous investigations have focused on the effects of histone modifications on gene promoters, whereas efforts to unravel their effects on transcribed regions have lagged behind. To elucidate the effects of histone modification on transcribed regions, we constructed a quantitative model, which we suggest can predict the variation of gene expression more faithfully than the model constructed on promoters. Moreover, motivated by the fact that exon spicing is functionally coupled to transcription, we also devised a quantitative model to predict alternative exon expression using histone modifications on exons. This model was found to be general across different exon types and even cell types. Furthermore, an interaction network linking histone modifications to alternative exon expression was constructed using partial correlations. The network indicated that gene expression and specific histone modifications (H3K36me3 and H4K20me1) could directly influence the exon expression, while other modifications could act in an additive way to account for the stability and robustness. In addition, our results suggest that combinations of histone modifications contribute to exon splicing in a redundant and cumulative fashion. To conclude, this study provides a better understanding of the effects of histone modifications on gene transcribed regions.

## Introduction

The expression of eukaryotic protein-coding genes is very complex. Transcription is carried out by RNA polymerase II (RNAPII), generating a primary RNA transcript (pre-mRNA). This must undergo a series of modifications to become a mature messenger RNA. These modifications include the addition of a 7-methyl guanosine cap at the 5′ end, 3′ end formation by cleavage and polyadenylation, and RNA splicing.

The eukaryotic genome is packaged in the form of nucleosomes, which are the basis of the chromatin structure. The histone components of the nucleosome undergo multiple post-translational covalent modifications including acetylation, methylation, phosphorylation and ubiquitination [1]–[2]. Chromatin packaging imposes an obstacle for protein binding to DNA as well as the processing and elongation of RNA polymerases [3]–[4]. Histone modifications can alter chromatin structure and act alone or jointly to facilitate numerous biological functions by changing the charge of the nucleosome particle, and/or by recruiting non-histone protein effectors [5]. Many types of histone modifications have been described, leading to the ‘histone code hypothesis’: specific combinations of histone modifications can result in distinct downstream effects [6]–[7]. However, others have proposed that histone modifications specify functions in cumulative rather than synergistic ways [8]–[10].

Links between gene expression and histone modifications have been established. Extensive studies show that histone acetylations are associated with gene activation [11]–[13]. Additionally, specific histone methylations such as H3K4me3 occur around the transcription start sites (TSS) of expressed genes and are associated with transcription initiation [14]–[15]. In particular, a recent study demonstrated that the histone modification levels in the promoter region are quantitatively correlated with the expression of the corresponding gene [16]–[17]. Many such studies focused on the effects of histone modifications on promoters, but investigations of the effects of such modifications along the transcribed region lagged behind. Gradually accumulated evidence suggests that histone modifications along the transcribed region might facilitate transcription elongation by RNAPII [18]–[21], which is also an essential step in gene regulation [22]. For example, H3K36me3, which accumulates toward the 3′ ends of genes, could regulate transcription elongation by enabling dynamic changes in chromatin compaction; H4K20me1 is also a marker of transcription elongation owing to its enrichment on the transcribed regions of active genes and sensitivity to specific elongation inhibitors. Furthermore, increasing evidence suggests that splicing is tightly coupled to transcription elongation. Pre-mRNA is spliced while it is still tethered to the DNA by RNAPII [23]–[25]. RNAPII can recruit many RNA splicing factors via its C-terminal domain (CTD) [23], [26]–[27]; the phosphorylated CTD of RNAPII interacts with the histone-lysine N-methyltransferase SETD2 [28]. Transcription elongation accompanies chromatin remodeling, which is frequently associated with histone modifications. Several chromatin remodelers have been shown to affect splicing by interacting with splicing factors and influencing the accumulation of RNAPII [29]–[30]. These facts suggest the possibility that histone modifications on transcribed regions might help regulate pre-mRNA splicing. In particular, a recent study has revealed that specific histone modification H3K36me3 could interact with polypyrimidine tract–binding protein (PTB) to regulate alternative splicing [31]. These facts elucidate the importance of histone modifications on transcribed regions, but the corresponding effect is still little understood.

In this paper, by analysis of genome-wide ChIP-seq datasets of histone modifications and the RNA-seq dataset in three human somatic cells, we derived a quantitative model for predicting gene expression using histone modifications on transcribed regions. Additionally, a quantitative model for predicting exon expression values was constructed using histone modification levels on constitutive and alternative exons, and this model was found to be general across different exon types and even different cell types. Furthermore, a network based on partial correlation analysis was constructed among histone modifications and exon expression. This method identified the histone modifications that contribute directly to exon inclusion with the transcription effect regressed away. The results implied that two specific histone modifications could directly influence exon expression, while other modifications could act in an additive way to account for the stability and robustness. We also studied the combinatorial effects of histone modifications, and investigated to what extent the histone code hypothesis is valid for splicing.

## Results

### Histone Modifications Along Transcribed Regions of Genes are Predictive for Gene Expression

Preliminary studies have indicated that histone modifications around promoters are predictive for expression of the corresponding gene [16]–[17]. Therefore, we examined whether there is a quantitative correlation between histone modifications along gene transcribed regions and the associated gene expression. The genome-wide ChIP-seq datasets of 38 histone modifications in human CD4+ T cells [32]–[33] and the RNA-seq dataset [34] were derived; the histone modification levels on gene transcribed regions and corresponding gene expression values were estimated (details are described in the Methods section). A simple linear regression model was used to relate the gene expression value to the histone modification levels, and the performance of the model was assessed by determining the Pearson correlation coefficients between predicted and measured expression. More sophisticated procedures have been applied to optimize the prediction, for example, in [16], different pseudo-counts were employed for the logarithmic transformations of different histone modifications to maximize their correlation with gene expression; alternatively, SVM regression [17] was used to improve linear regression. However, in this study only simpler procedures were borrowed to more clearly demonstrate and elucidate the quantitative relationship.

**Figure 1:**
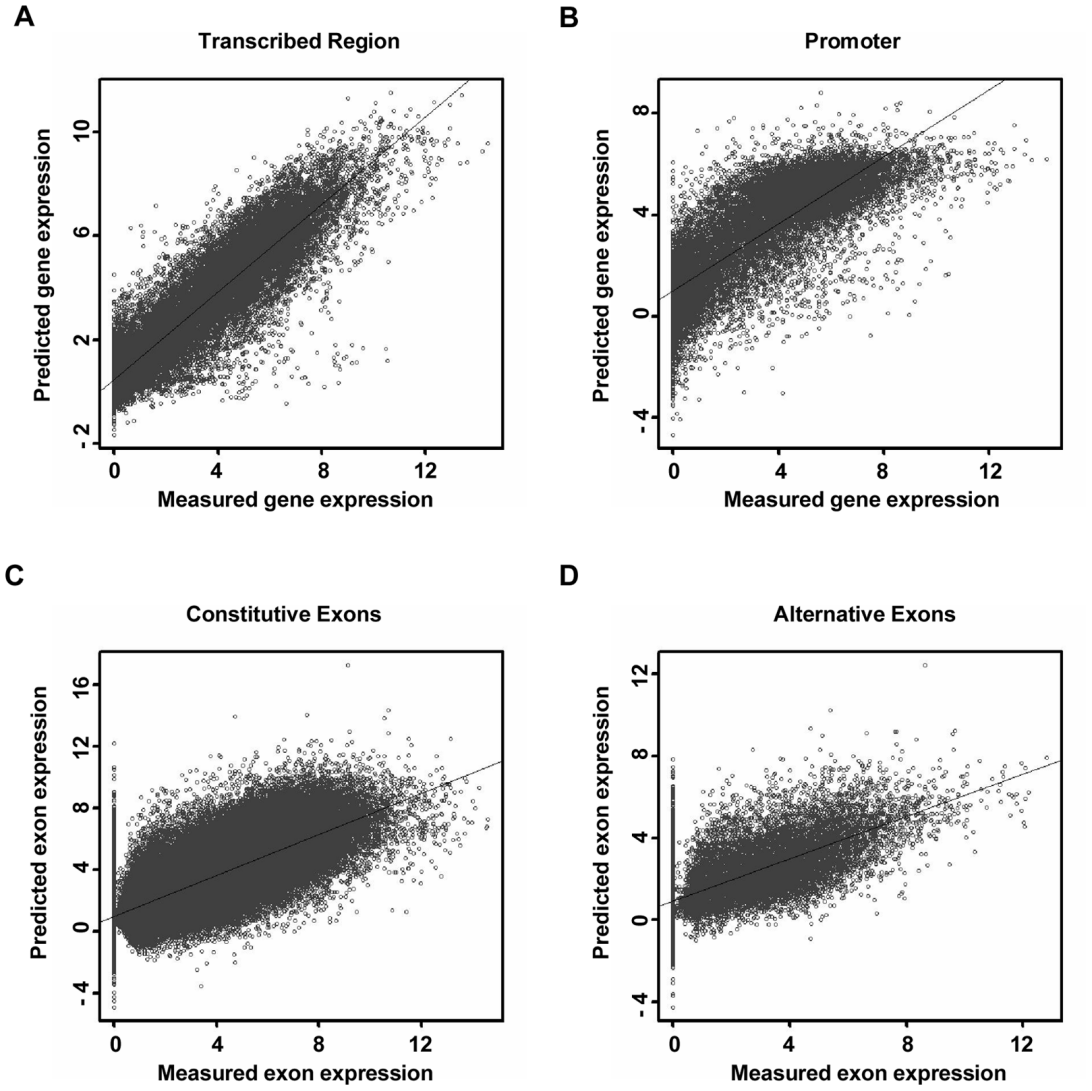
The quantitative correlation between histone modifications, gene expression and exon expression. The x axis represents the measured value of gene expression or exon expression. The y axis represents the predicted value by the linear regression model using the histone modification levels as input. (A-B) The scatterplots with predicted and measured gene expression values for transcribed regions and promoters. (C-D) The scatterplots with predicted and measured exon expression values for constitutive exons and cassette exons.

In Figure 1A, the Pearson correlation coefficient is 0.92 (p-value of t-test <2.2e-16), suggesting that histone modification levels along the transcribed regions were also strongly correlated with the gene expression values. Detailed information about the regression model can be found in Table S1. In addition, we shuffled the input order of histone modifications to ensure that none was in the right place, and then predicted gene expression; the Pearson correlation coefficients between predicted and measured expression values were all lower than 0.2. To compare the predictive power between transcribed regions and promoters, we repeated the experiments described in [16]. The Pearson correlation using the promoter model is 0.81 (Figure 1B), lower than that using the transcribed region model. Moreover, the comparison was repeated using ChIP-seq and RNA-seq datasets from two other cell types, the CD36+ T cell [35]–[36] and the H1 cell line [37] (Figure S1). In the CD36+ T cell, the Pearson correlation coefficients are 0.77 for the promoter model and 0.91 for the transcribed region model; in the H1 cell line, the Pearson correlation coefficients are 0.78 for the promoter model and 0.92 for the transcribed region model. These facts are consistent with our observations in the CD4+ T cell, suggesting that the histone modifications on transcribed regions might predict gene expression more faithfully.

In the model for transcribed regions, many histone modifications have significant regression coefficients (p-value corrected by Benjamini method [38]<0.001), and H3K36me3, H4K20me1 and RNAPII have the largest positive regression coefficients. The coefficients of the linear model could reveal the importance of histone modifications; that is, modifications with large positive coefficients probably function as activators while those with large negative coefficients probably correspond to suppressors. Accordingly, H3K36me3 and H4K20me1 along the transcribed regions could be crucial in regulating gene expression. Consistently, previous studies have observed that H3K36me3 and H4K20me1 are enriched along transcribed regions, and have indicated that these modifications could regulate RNAPII-catalyzed transcription elongation by enabling dynamic changes in chromatin compaction or collaborating with specific elongation inhibitors [18]–[21].

### Histone Modifications Along Exons are Predictive for Exon Expression

Splicing is functionally coupled to transcription [24], [39]–[41], raising the possibility that histone modifications might also contribute to the exon splicing. Having established a good quantitative correlation between gene expression values and histone modification levels on transcribed regions, we next asked whether there is also a correlation between exon expression values and histone modification levels along exons in transcribed regions.

The constitutive exons were first investigated, which are common to all mRNA transcripts for a given gene. A linear regression model and ten-fold cross-validation were employed again to characterize and confirm the quantitative relationship. Here, the explanatory variable is the exon expression value and the independent variables are the histone modification levels and RNAPII levels of the associated exons. As illustrated in Figure 1C, the Pearson correlation coefficient is 0.82 (p-value of t-test <2.2e-16). Further information about the regression can be found in Table S1.

Ideally, the constitutive exon is an indicator of gene transcription, and the expression levels of constitutive exons tend to be the same as the expression of the entire gene transcript. Thus, the above quantitative correlation is actually a correlation between histone modifications along constitutive exons and associated gene expression. Alternative splicing selectively includes or excludes RNA sequences, enabling individual genes to yield different transcripts [42]. If histone modifications were related to the splicing process, we would also expect to find a quantitative correlation between histone modifications along alternative exons and associated exon expression. To address this possibility, we considered the cassette exons (i.e. exons totally included or skipped). The exons with negative inclusion values were regarded as alternative splicing exons in the considered cell line (for details, see Methods). Linear regression and ten-fold cross-validation were employed to characterize and confirm the quantitative relationship. The good agreement illustrated in Figure 1D (r = 0.71, p-value of t-test <2.2e-16) suggests that the levels of histone modification along the alternative splicing exons are also well correlated with the expression values of the corresponding exons. In this linear regression model, H3K36me3 and H4K20me1 have the highest regression coefficients, suggesting a heavy influence of these two histone modifications in governing the exon expression levels. Consistently, recent studies have demonstrated significantly more H3K36me3 and H4K20me1 on exons than introns [43]–[44], and H3K36me3 has been validated as a regulator of alternative splicing [31].

Since exon expression is highly correlated with its associated gene expression, we needed to exclude the possibility that we were still modeling the correlation between gene expression and histone modifications. Therefore, the model for gene expression was used to predict the expression of cassette exons based on the histone modification levels on cassette exons. The accuracy was 0.63, which is about 12% lower than the model for cassette exons. This suggests that the relationship between histone modifications and gene expression could be different from that between histone modifications and exon expression.

### The Quantitative Correlation between Histone Modifications and Exon Expression is General

**Figure 2:**
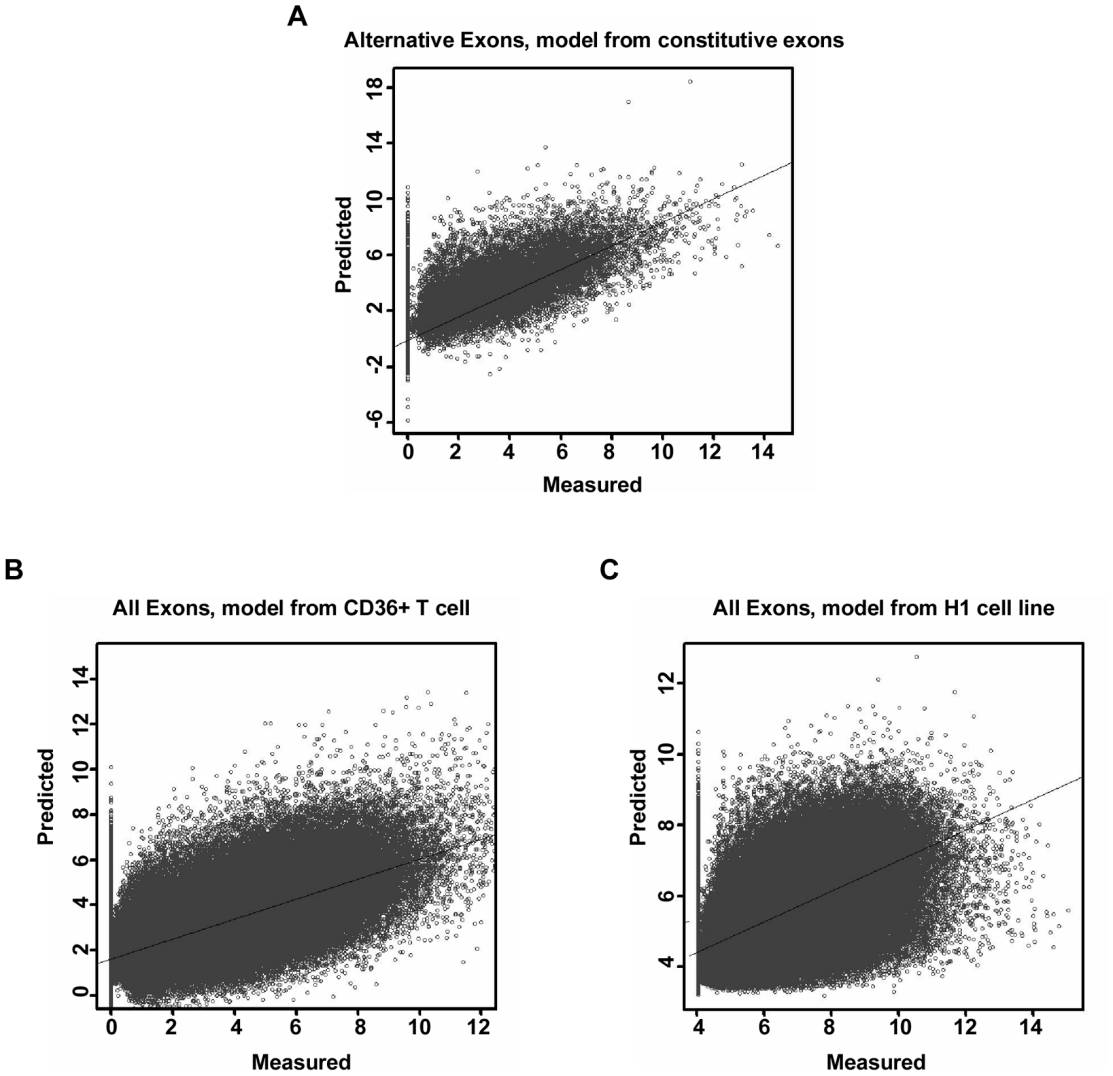
The quantitative relationship between histone modifications and exon expression is general. The x axis represents the measured value of exon expression. The y axis represents the predicted value by the linear regression model using histone modification levels as input. (A) The scatterplot with predicted and measured exon expression values for cassette exons. The linear regression model was trained on constitutive exons. On the basis of the resulting model, the histone modification levels on cassette exons were employed as input to predict the corresponding exon expression values. (B-C) The scatterplots with predicted and measured exon expression values in the CD4+ T cell. The linear regression models were trained on exons in the CD36+ T cell and H1 cell line, respectively. On the basis of the resulting models, the histone modification levels on exons of the CD4+ T cell were employed as input to predict the corresponding exon expression values.

Given the good quantitative correlations above, we next investigated whether this correlation is general. Firstly, the linear regression models constructed have demonstrated that histone modifications are quantitatively correlated with the expression of both constitutive and alternative exons. Furthermore, we assessed whether such quantitative correlations are shared by both kinds of exons. A linear regression model from constitutive exons was built and used to predict the expression of alternative exons (exon inclusion value <0). As illustrated in Figure 2A, the predicted and measured values are highly correlated (r = 0.71, p-value of t-test <2.2e-16 for alternative exons). In addition, the model from constitutive exons was used to predict the expression of exons whose exon inclusion values are less than −0.2, −0.4 and −0.5. The Pearson correlation coefficients were 0.69, 0.67 and 0.65, respectively (p-value of t-test <2.2e-16). These good agreements implied that the quantitative relationship between histone modification levels and exon expression values may be general and not limited to a specific kind of exon.

In addition, we investigated whether the resulting quantitative correlation between histone modifications and exon expression is limited to a specific cell type or is ubiquitous among cell types. First, a linear regression model was trained on the CD36+ T cell dataset, and next, according to the model obtained, the histone modification levels in the CD4+ T cell were used as input to predict the corresponding exon expression values. As shown in Figure 2B, the Pearson correlation coefficient between the predicted and measured expression values is 0.72 (t-test p-value<2.2e-16). However, it is worth noting that the exon expression levels in CD4+ and CD36+ T cells are similar. Therefore, to exclude the possibility that this similarity is responsible for the good prediction performance, we analyzed the exons whose expression levels differed significantly between the two cell types. The exons whose expression levels changed at least 2-fold, 5-fold and 10-fold between two cell types were selected. The regression model derived from the CD36+ T cell was used to predict the expression levels of these selected exons in the CD4+ T cell. The Pearson correlation coefficients between the predicted and measured exon expression levels were respectively 0.75,0.68 and 0.69 (Figure S2A-C). Moreover, a linear regression model was trained on H1 cell line datasets. According to this model, the histone modification levels in CD4+ T cells were then used as input to predict the corresponding exon expression values. As shown in Figure 2C, the Pearson correlation coefficient between the predicted and measured expression values was 0.65 (t-test p-value<2.2e-16). Also the exons whose expression values changed at least 2-fold, 5-fold and 10-fold between two cell types were selected. The corresponding Pearson correlation coefficients were respectively 0.65,0.63 and 0.62 (Figure S2D-F).

Thus, the results suggested that the quantitative correlation between histone modification levels and exon expression values could be universal across different exon types and even different cell types.

### An Interaction Network Among Histone Modifications and Cassette Exon Expression

A general quantitative relationship between histone modifications and exon expression has been presented above, but it cannot be interpreted as a direct interaction, since the quantitative correlation does not provide a way of distinguishing between direct and indirect associations. Some works have been reported to infer the relationship among histone modifications, non-histone proteins and gene expression [45]–[46], or the interplay among exon splicing, conserved sequence and splicing factors [47]. Those studies used clustering-based Bayesian network learning methods to recover the interaction relationships, but the clustering procedure might cause loss of information, and different procedures could yield different network structures [45]. In addition, the expression of alternative exons is to a great extent determined by the expression of the corresponding gene. Thus, to investigate whether a specific histone modification could result in differentiation between exon expression and gene expression, it is necessary to remove the transcription effect from exon expression. Gene-level-normalized exon intensity, which is defined as the ratio of exon expression to gene expression, has been widely used for studying alternative splicing. However, owing to the high-level of inherent noise, some studies using this approach have reported low validation rates for the identification of alternative splicing events [48]–[50]. Considering these facts, we applied the partial correlations to remove the transcription effect from exon expression and deduce the putative direct interaction between histone modifications and exon inclusion. Partial correlation has been widely utilized to model gene co-expression network and protein-protein interaction network [51]–[53]. A recent study employed partial correlation to study exon co-splicing networks, and achieved a higher statistical power than the approach based on gene-level-normalized exon intensity [54]. The partial correlation coefficient is the correlation that remains between two variables when the effects of the other variables are regressed away. For example, in order to exclude the possibility that a high correlation between one histone modification and exon expression is due to the association between that histone modification and gene expression, we calculated the partial correlation coefficient between the histone modification and exon expression conditional on gene expression. If the partial correlation remained high, it could be claimed that there is an association between the histone modification and exon expression and this association represents a putative direct regulatory relationship. In addition, the links between different histone modifications on exonic regions were studied, where a high correlation between two modifications is not due to their association with a third histone modification.

**Figure 3:**
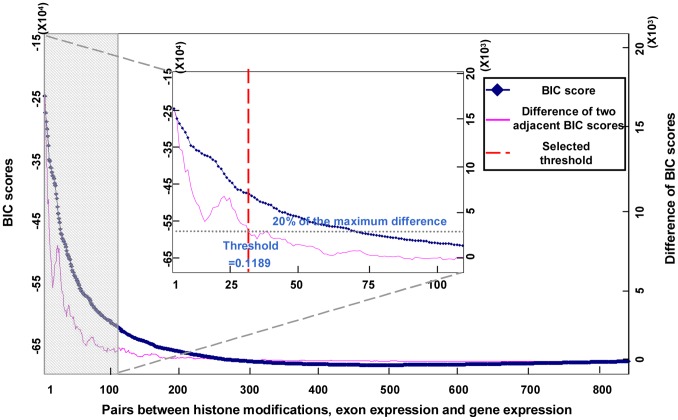
Threshold selection for the interaction network among histone modifications, gene expression and exon expression. The x axis represents the pairs between histone modifications, exon expression and gene expression. Such pairs were sorted by descending their partial correlations along x axis. The left y axis represents the BIC score, and the right y axis represents the difference in BIC score. Edges were successively assigned to the pairs along x axis, and the BIC scores for the newly-generated networks were calculated (blue line). Furthermore, the differences between two adjacent BIC scores were calculated (pink line). For clearer illustration, the shadowed part was enlarged into the figure in the middle. The grey dash indicated the 20% of the maximum difference, and the red dash indicated the pair between histone modifications with selected partial correlation threshold.

To derive such a model, we first measured the histone modification intensities on cassette exons, associated exon expression values and gene expression values. The partial correlation analysis was carried out directly with no data discretization, and thus avoided information loss as well as network structure instability resulting from different discretization methods. To eliminate indirect influences, each pair-wise partial correlation between histone modifications and exon expression was obtained conditional on all other histone modifications and gene expression. All pair-wise partial correlation coefficients are presented in Table S2. Next, a partial correlation threshold was selected to determine whether there is a regulatory relationship between two features, and to decide whether connections are assigned to such pairs. The choice of significance threshold remains a major challenge for network studies. Several approaches have been developed to select the threshold, such as the permutation procedure [55]–[56], expected FDR control [57] and scale-free topology criterion [58]. The FDR control and scale-free topology criterion methods are suitable for analyzing large-scale networks, but the histone modification interaction network is small. Therefore, a partial correlation threshold was selected based on the Bayesian information criterion (BIC), which accounts for the model complexity. The pairs between different histone modifications, exon expression and gene expression were sorted by descending their partial correlations. Next, the networks were generated by successively assigning edges to the sorted pairs, and then, the BIC score was calculated for each network. The blue line in Figure 3 indicates the BIC scores for different networks, and the pink line demonstrates the differences between two adjacent BIC scores. The BIC score was found to decrease continuously with the increasing model complexity, but it decreased only slightly after the interaction pair with partial correlation 0.1189. This suggests that the interactions with partial correlations greater than 0.1189 could be crucial for increasing the prediction accuracy of the whole model, so the partial correlation coefficient 0.1189 was chosen as threshold. Furthermore, the exon expression values and histone modification levels were permuted 100 times and a distribution of the new pair-wise partial correlations was recalculated for each permutation. The distribution of partial correlation coefficients is indicated in Figure S3. Permutation failed to create any associations with partial correlations over the selected threshold. The z-score of the threshold is 18.66 in this distribution. This fact suggests that the threshold found in the original dataset could not be generated by random chance. According to the selected threshold, the connections were assigned to the network. Thus, an undirected network for cassette exons was obtained, in which the nodes represent histone modifications and exon expression and the edges represent direct interactions between the connected pairs.

**Figure 4:**
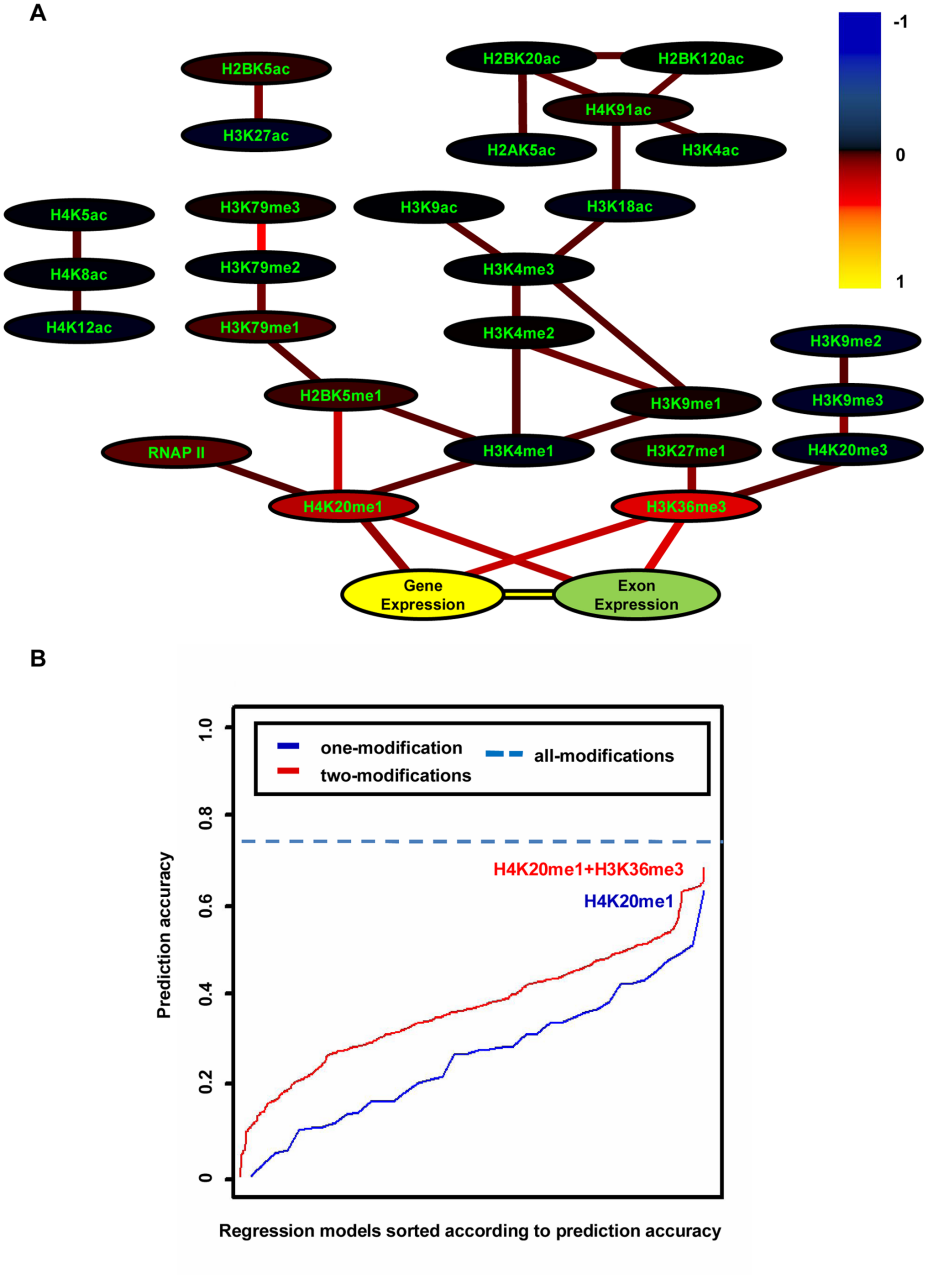
The interaction network among histone modifications, gene expression and exon expression. (A) To enable the maximum partial correlation coefficient to be 1.0, we normalized them by dividing each coefficient by the absolute value of the maximum coefficient. Colors were employed to represent the levels of normalized partial correlations. The colors of the edges denote the normalized partial correlations between connected nodes, and the colors of the nodes indicate the normalized partial correlations between corresponding nodes and the exon expression. Colors are scaled in the color legend. (B) The prediction accuracies by linear regression models constructed using all possible combinations of one (blue curve) and two (red curve) histone modifications. The models were sorted by ascending their prediction accuracies. The x axis denotes the combinations of one and two histone modifications. The y axis denotes the prediction accuracy. The dash line is the prediction accuracy of the full model by 38 histone modifications as well as RNAPII.

As demonstrated in Figure 4A, many histone modification interactions were observed, including the interactions between different levels of the same modification (H3K79me1, H3K79me2 and H3K79me3, together with H3K4me1, H3K4me2 and H3K4me3), between modifications on different amino acids (H3K4me1, H3K4me2 and H3K9me1), and between different kinds of modifications (H3K9ac and H3K4me3). In addition, two histone modifications (H3K36me3 and H4K20me1) and gene expression directly interacted with cassette exon expression and they all had positive partial correlations with exon expression. Some experiments described in the literature are consistent with our observations [20], [29]–[31]. However, it is worth noting that not both of the inferred histone modifications have the highest Pearson correlation coefficients with exon expression (see Table S2). To further validate such connections, we used all possible combinations of one and two histone modifications to predict the exon expression levels. It was expected that the inferred direct interactions would yield the most accurate prediction, even if not both of them have the highest Pearson correlation. Ten-fold cross-validation was employed to train and ascertain the models. As demonstrated in Figure 4B, the one-feature model comprising H4K20me1 and the two-feature model comprising H4K20me1 and H3K36me3 gave the highest prediction accuracies in their corresponding groups (Pearson correlation coefficients are 0.63 and 0.69, respectively). This fact to some extent confirmed that the interactions inferred by our method contribute directly to exon expression, whereas the other histone modifications could contribute indirectly to exon expression even if they have higher Pearson correlations with exon expression.

### Combinations of Histone Modifications Regulate Exon Expression in an Accumulative and Redundant Fashion

**Figure 5:**
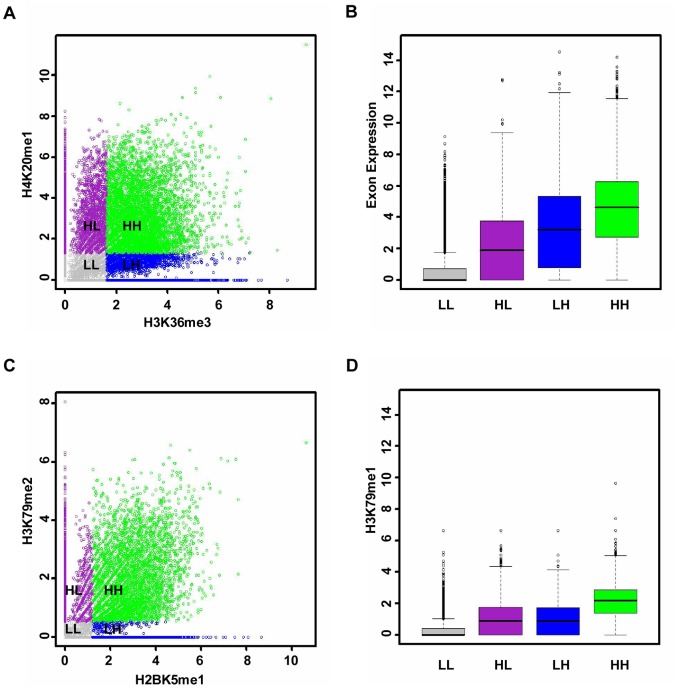
Co-regulation of histone modification combinations. (A and C) The x and y axis respectively represent the intensities of corresponding histone modifications. According to the histone modification intensities, all cassette exons are grouped into four bins: LL (grey), HL (purple), LH (blue) and HH (green). Whether the intensity is H or L is determined by comparing the histone modification intensity with the corresponding median values (1.63 for H3K36me3, 1.32 for H4K20me1, 1.21 for H2BK5me1 and 0.54 for H3K79me2). (B and D) The distributions of the exon expression and the intensity of H3K79me1 for the four bins.

Previous studies have explored the way in which the ‘histone code’ works. Some have hypothesized that histone modifications result in downstream effects in a synergistic fashion [6]–[7], while others have suggested that they specify functions in a collective manner [8]–[10]. Those studies focused on investigating how combinations of histone modifications on promoters regulate gene expression, but little is known about histone modifications on exon regions. We have obtained an interaction network among histone modifications and cassette exon expression. Furthermore, we assessed how such combinations on exons direct exon expression. Two combinations as examples were taken and an analytical approach similar to that in [17] was employed. Firstly, the network indicated that H3K36me3 and H4K20me1 interact directly with exon expression. All exons were then grouped into four bins according to the intensities of these two modifications. As demonstrated in Figure 5A-B, exon expression is lowest when both H3K36me3 and H4K20me1 are low, highest when both modifications are high and moderate when one modification is high and the other low. In addition, another combination (H2BK5me1 and H3K79me2) was considered, which interacts directly with histone modification H3K79me1. A significant difference was observed in H3K79me1 intensity from other bins when all of the modification intensities in the combination were high (Figure 5C-D). Similar phenomena were also observed in two other cell types CD36+ T cell and H1 cell line (Figure S4). These facts suggest that the combinations of histone modifications could regulate both exon expression and histone modifications in a cumulative rather than a synergistic fashion. To further validate this hypothesis, a multivariate regression model with interaction terms was performed (for details, see Methods). In the regression model (741 interaction terms), only six interactions are significant (Table S1, p-value corrected by Benjamini method [38]<0.001). Furthermore, to assess the importance of these interactions in determining exon expression levels, we compared the above regression model with a singleton model that contains no interaction terms. Ten-fold cross-validation was also utilized to evaluate the predictive power of the interaction model, and the Pearson correlation between predicted and measured exon expression levels was taken as the measure of its accuracy. It was found that the interaction model improves the accuracy of prediction by only 0.58% over the singleton model. The comparison was also repeated in the CD36+ T cell and H1 cell line. Consistent with the observations in the CD4+ T cell, the improvement ratios were still low (2.9% in the CD36+ T cell and 3.4% in the H1 cell line). The Pearson correlation coefficients are 0.70 and 0.71 for singleton models; 0.68 and 0.69 for interaction models. These facts suggest that the contribution of interactions to the prediction of exon expression is not substantial. Additionally, we performed the interaction regression models on all histone modification combinations predicted by the partial correlation network, and compared each of them with the corresponding singleton model. The interaction models did not make the accuracy of prediction significantly greater than the singleton models (Figure S5, the difference was 3% at most). The above facts to some extent validate the conclusion that combinations of histone modifications contribute to exon expression in a cumulative not a synergistic way.

**Figure 6:**
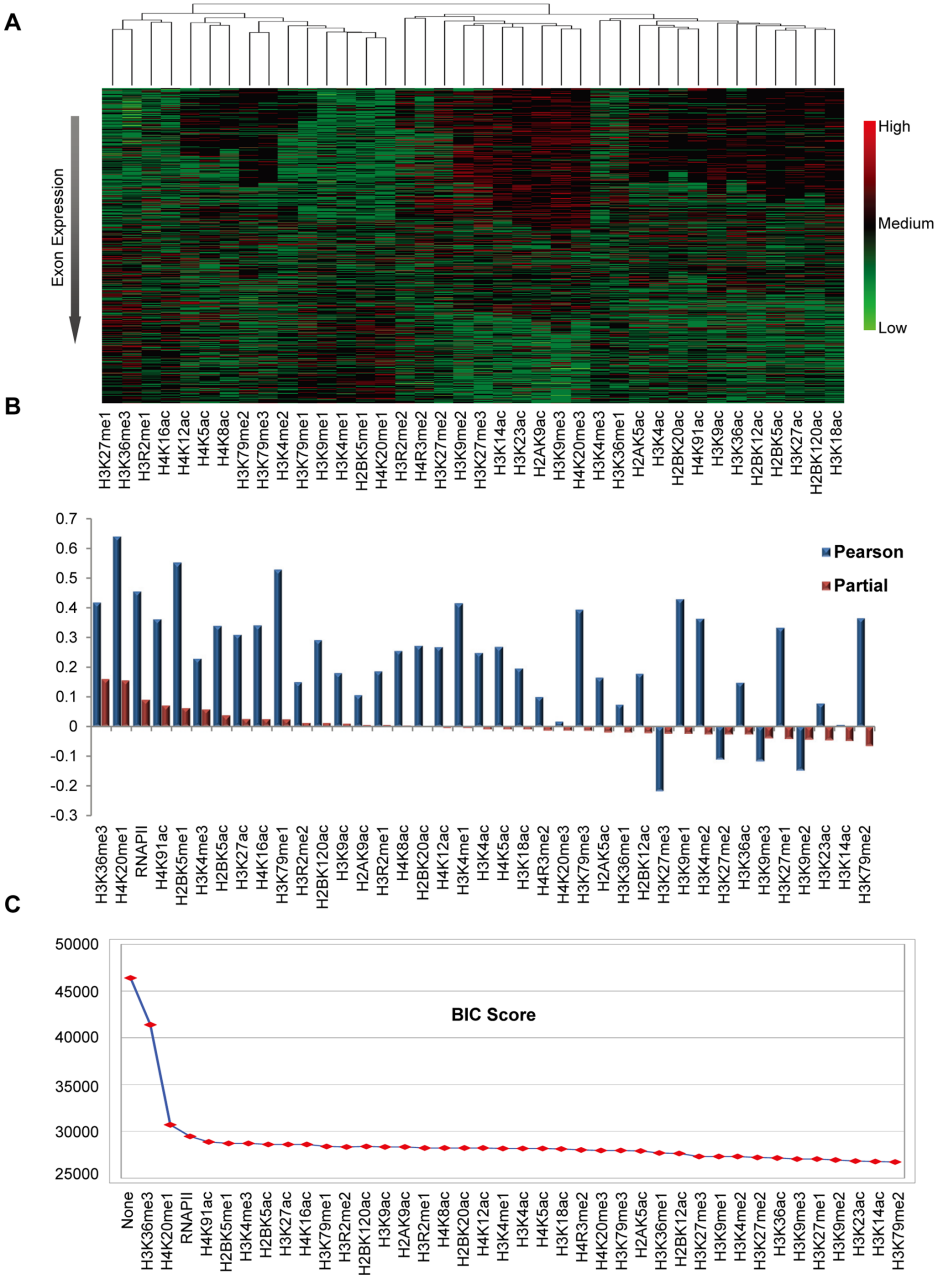
Redundancy exists among histone modifications. (A) The heatmap and hierarchical clustering for histone modifications on 17,713 alternative splicing exons. The exons were sorted according to the ascending expression levels from up to down. Hierarchical clustering was performed based on the signal profiles of histone modifications on all cassette exons. Colors are scaled in the color legend. (B) The Pearson correlation coefficients and partial correlation coefficients between histone modifications on exons and exon expression. (C) The plot of BIC scores for the linear regression models using different combinations of histone modifications. The y axis represents the BIC score. The x axis denotes the histone modification combination for the regression model. From left to right, the histone modification on x axis was added to the former combination. Then, the newly-generated combination was employed to construct a regression model and the corresponding BIC score was calculated.

In addition, we examined whether the redundancy exists among the histone modifications. In the unsupervised clustering (Figure 6A), different histone modifications exert similar intensity profiles within exons, implying that to some extent there exists redundancy among histone modifications. As demonstrated in Figure 6B, many histone modifications show high Pearson correlations with exon expression, but such correlations are not consistent with the trend of partial correlations. This also validates the existence of redundancy. Furthermore, the extent to which the redundancy exists was explored. As shown in Figure 5 and Figure S4, when both the histone modifications (H3K36me3 and H4K20me1) have high signals, exon expression is significantly higher, suggesting that histone modifications are not totally redundant. Finally, among the redundant histone modifications, the smallest combination was figured out, which could faithfully model exon expression. We added histone modifications successively to the prediction model according to the order of partial correlation (Figure 6B) and assessed the BIC score. The BIC score evaluates the prediction power of the histone modification combination, and meanwhile, penalizes the number of histone modifications. As shown in Figure 6C, the BIC value decreased monotonously, indicating that it is beneficial to include more histone modifications, even if the model complexity was penalized. However, it reduced only slightly after using the combination (H3K36me3 and H4K20me1). This fact suggests that such a combination could be good enough to model the variation of exon expression, and further validates their major role of in regulating exon splicing.

## Discussion

In this paper, a quantitative model for predicting the effects on gene expression of histone modifications on transcribed regions has been presented. We suggest that this model could capture the modulation of gene expression better than the model constructed on promoters. On this basis, a quantitative model for predicting alternative splicing exon expression was constructed using histone modifications on exons. Furthermore, an interaction network among histone modifications and cassette exon expression was constructed using partial correlations. This network indicated that exon expression is directly influenced by two factors: expression of the corresponding gene, and a specific combination of histone modifications (H3K36me3 and H4K20me1). It was also found that combinations of histone modifications contribute to exon expression in a redundant and cumulative fashion.

Recently, based on the genome-wide ChIP-seq and RNA-seq dataset in human CD4+ T cells, Karlic et al. [16] analyzed the quantitative relationship between histone modifications on promoters and gene expression. Similarly, Gerstein et al. [17] performed a more systematical analysis using the dataset generated by modENCODE [59]. Both of these methods utilized regression models to characterize the quantitative relationship between histone modification and gene expression (i.e., a simple regression model in [16] and a SVR model in [17]). However, these investigations have focused on the effects of histone modifications on gene promoters, while we assessed the effects of histone modifications along the transcribed regions on gene expression regulation. Our study was based on high-throughput sequencing datasets. To further validate the results, we referred to the related biological experiments in published literatures. On several candidate genes, Ref [60]–[62] utilized quantitative RT-PCR and ChIP to reveal the high distributions of H3K36me3, H4K20me1 and H3K27me3 along the transcribed regions, and indicated the correlation between these histone modifications and gene expression. These facts suggest that, not only the histone modifications on promoters, but also those on transcribed regions could affect gene expression. Moreover, we found that the model for transcribed regions gives even more accurate predictions than the model built on promoters [16], implying that histone modifications on transcribed regions could more directly account for the variation of gene expression than those on promoters.

Exon splicing and transcription elongation are traditionally thought to function separately, one at the RNA level, and the other at the DNA level, but increasing evidence suggests that splicing is functionally coupled to transcription elongation [25]. On this basis, a linear regression model for predicting exon expression was built using histone modifications on constitutive as well as alternative splicing exons. A high prediction accuracy was obtained, revealing a close association between histone modifications and exon expression. Consistently, based on quantitative RT-PCR and ChIP, Ref [31], [63] found that the distributions of a set of histone modifications on alterative spliced exons, including H3K36me3, H3K4me1, H3K27me3, H3K4me3 and H3K9me1, significantly changed accordingly with the differential exon expression in different cell types. These biological experiments further verified our founding. Recently, a rule-based model was trained to predict exon inclusion levels (high or low) from the histone modification combinations [64]. The generated model is in the form of “IF … THEN …”, and had a high prediction accuracy. As well as this work, that study also implies that histone modifications along transcribed regions could play a role in regulating exon splicing. However, that study suggests that such role is in a switch on/off way, while the results here revealed a continuous way with levels of histone modifications rising or falling over a large range of expression values. Furthermore, this relationship was found to be general across different exon types and different cell types. This enables us to declare the relationship between histone modifications on exons and associated exon expression with more confidence.

To further explore the relationship among histone modifications, gene expression and exon expression, we constructed an interaction network based on partial correlations. The network indicated that aside from gene expression, two histone modifications (H3K36me3 and H4K20me1) contribute directly to exon expression. This suggests that such two histone modifications could account for exon inclusion with transcription effects regressed away. Consistently, Luco et al. [31] utilized biological experiments (RNA interference, RNA immunoprecipitation and quantitative RT-PCR) to validate the causal role of histone modifications in alternative splice site selections, and indicated that H3K36me3 could interact with PTB protein to regulate alternative splicing. A preliminary study by Han et al. [45] utilized a Bayesian network to infer the histone modification regulatory network on gene promoters, while this work investigates the histone modification interaction relationships on exons. A recent study [65] indicated that the cross-talk between histone modifications could evolve over different genomic elements. Consistently, Zippo et al. [66] found that if a histone phosphorylation H3S10ph respectively happens on the enhancer and promoter, it will induce different histone modifications, and consequently, result in different gene expression status. Compared to the network on promoters [45], different histone modification interactions were actually revealed on exons. This possibly results from three mechanisms. Firstly, promoters and exons could recruit different protein complexes. Wang et al. [67] revealed distinct distributions of histone acetyltransferase and deacetyltransferase on promoters and transcribed regions, including p300, CBP, PCAF, MOF, Tip60, HDAC1, HDAC2, HDAC3 and HDAC6. These distinct distributions could contribute to the different histone modification interaction relationships on promoters and exons. Secondly, the specific sequence features on two different genomic elements could result in the different regulatory relationships. For example, compared to promoters, exons have specific sequence Exon Splicing Silencer (ESS). Luco et al. [31] indicated that ESS could recruit PTB; furthermore, PTB interacts with MRG15, a component of the retinoblastoma binding protein 2 (histone demethylase complex) [68]. Thirdly, genomic elements could exert different chromatin structure, which associate with different chromatin remodeling proteins. Some chromatin remodelers have been indicated to function as or associate with histone modifiers [69]–[70].

In the partial correlation interaction network, specific combinations of histone modifications were identified, some contribute directly to exon expression and others regulate the differentiation of histone modification levels. Traditionally, proponents of the histone code hypothesis have held that specific combinations of histone modifications specify distinct downstream effects in a synergistic fashion [6]–[7], while other studies have only revealed the simple additive consequence of histone modifications [8]–[10]. Consistently with the latter hypothesis, our results suggested that combinations of histone modifications on exons regulate exon expression cumulatively rather than synergistically. In addition, the existence of redundancy was observed in the histone modifications on exon regions. Our results suggested that only two histone modifications (H3K36me3 and H4K20me1) could faithfully predict exon expression levels. However, the redundant histone modifications cannot be considered meaningless, since they could act in additive ways and the multitude of modifications could contribute to stability and robustness [71], therefore indirectly contributing to exon splicing. The remaining modifications might also stabilize the major modifications through feedback loops and mutual interactions.

The quantitative predictive model and the partial correlation network were constructed among histone modifications and exon expression, but it is very difficult to identify the directions of the connections. In fact, both directions of causality have been reported. Luco et al. suggest that histone modification could interact with splicing factors to influence alternative splicing [31], while a recent study by Kim et al. indicates that specific histone modifications could be determined by pre-mRNA splicing [63]. Moreover, there is bidirectional causality among histone modifications, and such feedback loops could be important for making the interaction network robust and stable [71]. Therefore, the cause-effect relationships need to be elucidated by further incorporating different types of data.

Finally, these results offer a better understanding of the roles of histone modifications on transcribed regions. With the generation of more and more high-throughput sequencing datasets of novel histone modifications, the significance of such modifications needs to be studied. Our study presents a tool for investigating the relationship between histone modifications and exon expression. Molecular experiments are still required to investigate this relationship, but our study could help to reduce the range of experimental investigations required. Additionally, our results provide a framework for predicting exon expression levels using histone modifications.

### Conclusion

To elucidate the effects of histone modification on transcribed regions of genes, this paper constructed a quantitative model for predicting gene expression using histone modifications on such regions, and suggested that it captures the variation of gene expression more faithfully than the model constructed on promoters. Moreover, motivated by the fact that exon spicing is functionally coupled to transcription, we constructed a quantitative model for predicting alternative exon expression using histone modifications on exons, and demonstrated that it differs from the model for gene expression. Furthermore, a partial correlation network was employed to identify histone modifications that directly influence exon expression with the transcription effect removed. In addition, the way in which the ‘histone code’ on exonic regions contributes to exon splicing was explored. Thus, our results provide a better understanding of the roles of histone modifications on gene transcribed regions.

## Materials and Methods

### Data Sets

Genome-wide ChIP-seq data of 38 histone modifications of human CD4+ T cells and ChIP-seq data for RNAPII were obtained from [32]–[33]. The RNA-seq dataset of human CD4+ T cells was downloaded from [34]. We also derived the human RefGene annotation from the UCSC genome browser [72] to determine the genomic coordinates of the sequences in the ChIP-seq data, and obtained the KnownAlt table to identify the annotation of alternative splicings. All read lengths were extended to 100 base pairs in the direction of the strand to which they map.

### Obtaining Histone Modification Levels, Exon Expression Values and Gene Expression Values from ChIP-seq and RNA-seq Datasets

The gene location sites were obtained from the RefGene annotation. The RNA-seq reads and ChIP-seq reads of 38 histone modifications as well as RNAPII were aligned to RefGenes and summed. In order to produce a measurement of expression value normalized to the length of the gene, the sums of RNA-seq reads mapping to genes were divided by the number of base pairs in exonic regions. Likewise, the sums of histone modification ChIP-seq reads were divided by the number of base pairs in exonic regions, since there are significantly more nucleosomes - in which histone modifications take place - in exons than in introns. We added a pseudo-count of 0.01 to the length-normalized read sums to avoid undefined values of the logarithm, and treated the corresponding logarithmic transformations as the estimated histone modification levels on transcribed regions and measured gene expression values.

In addition, on the basis of the KnownAlt table, we eliminated all putative alternative splicing exons from the exons obtained from RefGene, thus deriving the constitutive exon annotations. The location site annotations of cassette exons (i.e. exons totally included or skipped) were derived from the table KnownAlt. The fold change (log2) was calculated between exon expression and the corresponding gene expression, and was treated as the indicator of exon inclusion value. Exons with negative inclusion values were regarded as alternative splicing exons. The first and last exons in a given gene were not considered, since they could be influenced to a great extent by transcription. RNA-seq reads and ChIP-seq reads of histone modifications as well as RNAPII were then aligned to exons and summed. The sums of reads mapping to exons were divided by the number of base pairs in the associated exon to produce a measurement of expression value and histone modification levels normalized to exon length. A pseudo-count of 0.01 was added to these length-normalized sums. The logarithms of these normalized read counts were taken as the measurements of exon expression and the histone modification intensities on exons.

The procedure in [16] was repeated to assess the levels of histone modifications on promoters. ChIP-seq reads of histone modifications were aligned to a window of 4,001 base pairs surrounding the TSS of each gene, and the logarithmic transformation of the read sum was taken as an estimate of the levels of histone modification on the promoters.

### Linear Regression Model

To build the quantitative correlation between histone modification intensities on transcribed regions of genes and associated gene expression, a linear regression model was constructed, which is described as follows:
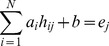
(1)


where *N* represents the number of chromatin features; 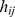
is the independent variable, representing intensity of histone modification *i* on transcribed region of gene *j*; 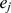
is the explanatory variable, representing the expression of gene *j*; and 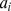 and *b* are the slope and intercept of the linear regression model, respectively. The t-test was utilized to evaluate the significance of regression coefficients and Benjamini method [38] was utilized to address the multiple testing issue.

Ten-fold cross-validation confirms the quantitative relationship. The dataset of histone modification intensities and associated gene expression values was randomly grouped into 10 bins. Nine bins were used to train the linear model, and then on the basis of the resulting model, the histone modification levels of the remaining genes were used as input to predict their expression. This procedure was repeated ten times. The performance of the model was evaluated by determining the average of the Pearson correlation coefficients between measured expression and corresponding predicted expression in 10 non-overlapping bins. The procedures for construction and confirmation of linear regression models for histone modification levels on exons and corresponding exon expression values were all similar to the above.

In addition, a multivariate regression model with interaction terms was performed to investigate the interactions among histone modifications. The model is shown as follows:
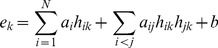
(2)where *N* represents the number of chromatin features; *h_ik_* is the independent variable, representing the intensity of the *i^th^* histone modification on the *k^th^* exon; *e_k_* is the explanatory variable, representing the expression level of the *k^th^* exon; and *a* and *b* are the slope and intercept of the linear regression model, respectively.

A significant interaction term in this model would imply that the interaction between two histone modifications has a significant effect on exon expression. Ten-fold cross-validation was also utilized to evaluate the predictive power of the interaction model, and the Pearson correlation between predicted and measured exon expression levels was taken as the measure of its accuracy. Furthermore, this model was compared with the singleton model: 
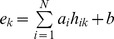
. The improvement ratio was calculated as: (P_singleton_-P_interaction_)/P_singleton_, where P_singleton_ and P_interaction_ are the Pearson correlation coefficients between measured and predicted values for the singleton model and interaction model, respectively. Additionally, interaction regression models were performed on all histone modification combinations in the partial correlation network. For example, the network indicated that H3K4me1, H3K79me1 and H4K20me1 contribute to H2BK5me1 in a combinatorial way, therefore, an interaction regression model was constructed as follows: H2BK5me1 = *a_1_*×H3K4me1+ *a_2_*×H3K79me1+ *a_3_*×H4K20me1+ *a_4_*×H3K4me1× H3K79me1+ *a_5_*×H3K4me1×H4K20me1+ *a_6_*×H3K79me1×H4K20me1+*b.* There are eighteen similar combinations of histone modifications. Thus, we constructed eighteen interaction regression models and compared each of them with the corresponding singleton model.

### Partial Correlation Coefficient

The R function pcor.test (http://www.yilab.gatech.edu/pcor.html) was used to calculate the partial correlation coefficients. The partial correlation coefficient *P_AB,C_* between variables *A* and *B*, conditional on another variable *C* is described as follows. *A* and *B* are hypothesized to be linearly related to *C*.
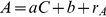
(3)

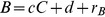
(4)


The partial correlation coefficient *P_AB,C_* is defined as the correlation coefficient between residuals *r_A_* and *r_B_*. The partial correlation coefficient *P_AB,C_* between *A* and *B* conditional on *C* can be computed as follows:
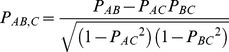
(5)where *P_AB_*, *P_BC_* and *P_AC_* respectively represent the pair-wise correlations between variables *A*, *B* and *C*. The value of *P_AB,C_* is between −1 and 1.

### Partial Correlation Network

The partial correlation network is a graph, 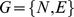, where the node set, *N*, are the variables of interests (here, they are histone modifications and exon expression); the edge set, *E*, is the interaction between variables. *e_AB_* is an edge in *G*, if 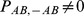, where *P_AB,-AB_* is the partial correlation coefficient between variables A and B, conditional on all other variables except *A* and *B*.

From the statistical aspect, the partial correlation network is one kind of conditionally independent network [51]. Variables *A* and *B* are connected in the partial correlation network if there is no other variable for which *A* and *B* are conditionally independent. From the geometrical aspect, the partial correlation network is equivalent to a proximity graph of variables [51], and more concretely, it is a 'Gabriel graph' [73]. *A* and *B* are connected in the Gabriel graph if no other point falls within the sphere that has the chord *A*, *B* as its diameter.

### Network Construction, Threshold Selection and Permutation Test for Significance

To derive such a model, we first used the procedure above to obtain the histone modification intensities on exons, associated exon expression values, and gene expression values. Secondly, each pair-wise partial correlation was calculated among histone modifications, exon expression and gene expression conditional on all other variables to remove the indirect influence. Thirdly, a threshold was selected to assess whether the partial correlation was significant. If the partial correlation was higher than the threshold, an edge was assigned to the pair. For this work the threshold was selected based on the BIC criterion, which assesses a model by penalizing its complexity and evaluating the goodness of fit between sample data and the model. The BIC criterion is described as follows:
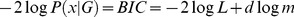
(6)


where *x* represents samples for histone modifications and exon expressions; *G* is the estimated model; *L* is the maximized value of the likelihood function 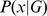; *m* is the sample size of *x*; *d* is the number of free parameters; and herein, *d* is the number of regressors of the linear regression model, including the intercept.

To select the threshold, we first sorted the pairs between different histone modifications, exon expression and gene expression (41×40/2 = 820 pairs in the network) by descending their partial correlations. Next, 820 networks were generated by successively assigning edges to the sorted pairs, and then the BIC score was calculated for each network. As long as the BIC score decreases, it is beneficial to add new edges. However, to preclude the addition of too many edges to the network, we kept only the crucial interactions. Assigning edges to these crucial interactions would significantly reduce the BIC score of the network. Therefore, we selected a threshold from which the BIC score changed slightly. The difference in the BIC score was calculated as *BIC_i_*_-1_-*BIC_i_*, where *BIC_i_* represents the score for the *i^th^* network. The difference helped to determine the extent to which the BIC score changes. The BIC score is assumed to change over a small scale, when the corresponding difference is less than 20% of the maximum difference.

Furthermore, to examine whether the selected threshold can be expected randomly, we independently permuted the datasets of the histone modification intensities on exons and associated exon expression values to enable that no single data corresponds to the right exon in each dataset. The partial correlation coefficients between different histone modifications and exon expression were calculated based on the generated datasets. The permutation was repeated 100 times, and a distribution of the new pair-wise partial correlations was recalculated for each permutation.

According to the threshold, the connections were assigned to the network. It is an undirected network.

### Predicting Exon Expression Across Cell Types

The ChIP-seq datasets of eight histone modifications, H3K4me1, H3K4me3, H3K27me1, H3K27me3, H4K20me1, H3K9me1, H3K9me3 and H3K36me3, as well as RNAPII in the human CD36+ T cell, were obtained from [35]. The RNA-seq dataset was derived from Gene Expression Omnibus (GSE26501) [36]. In addition, the ChIP-seq datasets of twenty histone modifications, H3K4me1, H3K4me2, H3K4me3, H3K9me3, H3K36me3, H3K79me1, H3K79me2, H4K20me1, H2AK5ac, H2BK5ac, H2BK12ac, H2BK20ac, H2BK120ac, H3K4ac, H3K9ac, H3K18ac, H3K23ac, H3K27ac, H4K5ac, H4K91ac and the RNA-seq dataset in the H1 cell line were obtained from NIH Epigenomics Roadmap Consortium [37]. Histone modifications and RNAPII shared by both CD4+ T cell and CD36+ T cell as well as those by both CD4+ T cell and H1 cell line were considered in this analysis. The histone modification levels and expression value for each exon were obtained as described above. To normalize the exon expression values and histone modification levels relative to the corresponding values in the CD4+ T cell, we derived a regression line between the corresponding values and then modified the values in the CD36+ T cell and H1 cell line to transform the regression line into *y = x*. To check whether the quantitative correlation was general across cell types, we first trained linear regression models using the normalized histone modification levels and associated exon expression in the CD36+ T cell and the H1 cell line, and then used the histone modification levels in the CD4+ T cell as input to predict the associated exon expression. The Pearson correlation coefficient between the predicted and measured exon expression values in the CD4+ T cell was calculated.

### Comparison between the Prediction Capabilities of Linear Regression Models using Different Histone Modification Combinations

All possible one-modification and two-modification combinations were generated. The linear regression model was trained for each combination of histone modifications, where levels of histone modifications in each combination are independent variables, and the corresponding exon expression value is the explanatory variable. Ten-fold cross-validation was employed, and the Pearson correlation coefficient between predicted values and measured values was treated as the performance evaluation of the linear regression model for a given combination of histone modifications.

## Supporting Information

Figure S1 **Histone modifications along transcribed regions predict gene expression more faithfully than those on promoters.** The x axis represents the measured value of gene expression. The y axis represents the predicted value by the linear regression model using the histone modification levels as input. (A-B) The scatterplots with predicted and measured gene expression values for transcribed regions and promoters in the CD36+ T cell. (C-D) The scatterplots with predicted and measured gene expression values for transcribed regions and promoters in the H1 cell line.
(TIF)

Figure S2 **Prediction of exon expression across cell types.** The x axis represents the measured value of exon expression. The y axis represents the predicted value by linear regression model using histone modification levels as input. (A-C) The prediction of exon expression in the CD4+ T cell using the linear regression model built from the CD36+ T cell. The linear regression model was trained on exons in the CD36+ T cell. Based on the resulting model, the histone modification levels on exons of the CD4+ T cell were employed as input to predict the corresponding exon expression values. This analysis was performed on exons whose expression changed at least 2-fold (A), 5-fold (B) and 10-fold (C) between two cell types. (D-F) The prediction of exon expression in the CD4+ T cell using the linear regression model built from the H1 cell line. The linear regression model was trained on exons in the H1 cell line. Based on the resulting model, the histone modification levels on exons of the CD4+ T cell were employed as input to predict the corresponding exon expression values. This analysis was performed on exons whose expression changed at least 2-fold (D), 5-fold (E) and 10-fold (F) between two cell types.
(TIF)

Figure S3 **The significance of the selected threshold was validated using the permutation test.** The histogram for the pair-wise partial correlations of the permutation. It illustrates the frequencies of partial correlation coefficients for the permutation. The blue and red triangles respectively represent the maximum partial correlation coefficient generated by permutation and the selected threshold for the interaction network.
(TIF)

Figure S4 **Co-regulation of histone modification combinations in the CD36+ T cell and H1 cell line.** (A and C) The x and y axis respectively represent the intensities of corresponding histone modifications. In the CD36+ T cell and H1 cell line, all exons were respectively grouped into four bins: LL (grey), HL (purple), LH (blue) and HH (green). Whether the intensity is H or L is determined by comparing the histone modification intensity with the corresponding median value (1.43 for H3K36me3 and 1.16 for H4K20me1 in the CD36+ T cell; 1.77 for H3K36me3 and 1.91 for H4K20me1 in the H1 cell line). (B and D) The distributions of the exon expression for the four bins in two cell types.
(TIF)

Figure S5 **The interaction model did not lead to obvious improvement compared to the singleton model.** Singleton and interaction regression models were respectively constructed according to eighteen histone modification combinations indicated by the partial correlation network. The x axis denotes the explanatory variables in eighteen regression models. The y axis represents Pearson correlation coefficients between measured and predicted values. The explanatory variables were sorted by ascending Pearson correlation coefficients along the x axis.
(TIF)

Table S1 Detailed information of the linear models. This file is.xls format. This table respectively presents the detailed information of the linear model for histone modification levels on transcribed regions and gene expression values, the linear model for histone modification levels on constitutive exons and associated exon expression values, the linear model for histone modification levels on cassette exons and associated exon expression values as well as the interaction regression model for histone modification levels on cassette exons and associated exon expression values. For each model, this table respectively presents the regression coefficients, standard errors, t-statistics and corresponding p-values corrected by Benjamini method. The histone modifications marked in yellow have the largest regression coefficients. The interaction terms marked in red have significant regression coefficients (p-value<0.001).
(XLS)

Table S2 The pair-wise Pearson and partial correlation coefficients between histone modifications and cassette exon expression. This file is.xls format. The values marked in yellow represent the partial correlations between histone modifications and cassette exon expression. The values marked in green represent the Pearson correlations between histone modifications and exon expression. The values marked in red suggest that there exists an interaction between this pair.
(XLS)

## References

[1] Bernstein BE, Meissner A, Lander ES (2007) The mammalian epigenome. Cell 128: 669–681. 17320505 10.1016/j.cell.2007.01.033

[2] Kouzarides T (2007) Chromatin modifications and their function. Cell 128: 693–705. 17320507 10.1016/j.cell.2007.02.005

[3] Wasylyk B, Chambon P (1979) Transcription by eukaryotic RNA polymerases A and B of chromatin assembled in vitro. Eur J Biochem 98: 317–327. 226362 10.1111/j.1432-1033.1979.tb13191.x

[4] Izban MG, Luse DS (1991) Transcription on nucleosomal templates by RNA polymerase II in vitro: inhibition of elongation with enhancement of sequence-specific pausing. Genes Dev 5: 683–696. 2010092 10.1101/gad.5.4.683

[5] Tse C, Sera T, Wolffe AP, Hansen JC (1998) Disruption of higher-order folding by core histone acetylation dramatically enhances transcription of nucleosomal arrays by RNA polymerase III. Mol Cell Biol 18: 4629–4638. 9671473 10.1128/mcb.18.8.4629 PMC109049

[6] Strahl BD, Allis CD (2000) The language of covalent histone modifications. Nature 403: 41–45. 10638745 10.1038/47412

[7] Jenuwein T, Allis CD (2001) Translating the histone code. Science 293: 1074–1080. 11498575 10.1126/science.1063127

[8] Dion MF, Altschuler SJ, Wu LF, Rando OJ (2005) Genomic characterization reveals a simple histone H4 acetylation code. Proc Natl Acad Sci U S A 102: 5501–5506. 15795371 10.1073/pnas.0500136102 PMC555684

[9] Schubeler D, MacAlpine DM, Scalzo D, Wirbelauer C, Kooperberg C, et al (2004) The histone modification pattern of active genes revealed through genome-wide chromatin analysis of a higher eukaryote. Genes & Development 18: 1263–1271. 15175259 10.1101/gad.1198204 PMC420352

[10] Kurdistani SK, Tavazoie S, Grunstein M (2004) Mapping global histone acetylation patterns to gene expression. Cell 117: 721–733. 15186774 10.1016/j.cell.2004.05.023

[11] Liang G, Lin JC, Wei V, Yoo C, Cheng JC, et al (2004) Distinct localization of histone H3 acetylation and H3-K4 methylation to the transcription start sites in the human genome. Proc Natl Acad Sci U S A 101: 7357–7362. 15123803 10.1073/pnas.0401866101 PMC409923

[12] Kuo MH, Allis CD (1998) Roles of histone acetyltransferases and deacetylases in gene regulation. Bioessays 20: 615–626. 9780836 10.1002/(SICI)1521-1878(199808)20:8<615::AID-BIES4>3.0.CO;2-H

[13] Shahbazian MD, Grunstein M (2007) Functions of site-specific histone acetylation and deacetylation. Annu Rev Biochem 76: 75–100. 17362198 10.1146/annurev.biochem.76.052705.162114

[14] Kouzarides T, Santos-Rosa H, Schneider R, Bannister AJ, Sherriff J, et al (2002) Active genes are tri-methylated at K4 of histone H3. Nature 419: 407–411. 12353038 10.1038/nature01080

[15] Bernstein BE, Humphrey EL, Erlich RL, Schneider R, Bouman P, et al (2002) Methylation of histone H3 Lys 4 in coding regions of active genes. Proc Natl Acad Sci U S A 99: 8695–8700. 12060701 10.1073/pnas.082249499 PMC124361

[16] Karlic R, Chung HR, Lasserre J, Vlahovicek K, Vingron M (2010) Histone modification levels are predictive for gene expression. Proc Natl Acad Sci U S A 107: 2926–2931. 20133639 10.1073/pnas.0909344107 PMC2814872

[17] Cheng C, Yan KK, Yip KY, Rozowsky J, Alexander R, et al.. (2011) A statistical framework for modeling gene expression using chromatin features and application to modENCODE datasets. Genome Biology 12. 10.1186/gb-2011-12-2-r15 PMC3188797 21324173

[18] Vakoc CR, Sachdeva MM, Wang H, Blobel GA (2006) Profile of histone lysine methylation across transcribed mammalian chromatin. Mol Cell Biol 26: 9185–9195. 17030614 10.1128/MCB.01529-06 PMC1698537

[19] Bell O, Wirbelauer C, Hild M, Scharf AN, Schwaiger M, et al (2007) Localized H3K36 methylation states define histone H4K16 acetylation during transcriptional elongation in Drosophila. EMBO J 26: 4974–4984. 18007591 10.1038/sj.emboj.7601926 PMC2140113

[20] Talasz H, Lindner HH, Sarg B, Helliger W (2005) Histone H4-lysine 20 monomethylation is increased in promoter and coding regions of active genes and correlates with hyperacetylation. Journal of Biological Chemistry 280: 38814–38822. 16166085 10.1074/jbc.M505563200

[21] Briggs SD, Du HN, Fingerman IM (2008) Histone H3 K36 methylation is mediated by a trans-histone methylation pathway involving an interaction between Set2 and histone H4. Genes & Development 22: 2786–2798. 18923077 10.1101/gad.1700008 PMC2569878

[22] Shilatifard A, Krogan NJ, Dover J, Wood A, Schneider J, et al (2003) The Paf1 complex is required for histone h3 methylation by COMPASS and Dot1p: Linking transcriptional elongation to histone methylation. Molecular Cell 11: 721–729. 12667454 10.1016/s1097-2765(03)00091-1

[23] de Almeida SF, Carmo-Fonseca M (2008) The CTD role in cotranscriptional RNA processing and surveillance. FEBS Lett 582: 1971–1976. 18435923 10.1016/j.febslet.2008.04.019

[24] Allemand E, Batsche E, Muchardt C (2008) Splicing, transcription, and chromatin: a menage a trois. Curr Opin Genet Dev 18: 145–151. 18372167 10.1016/j.gde.2008.01.006

[25] Moore MJ, Proudfoot NJ (2009) Pre-mRNA processing reaches back to transcription and ahead to translation. Cell 136: 688–700. 19239889 10.1016/j.cell.2009.02.001

[26] Howe KJ (2002) RNA polymerase II conducts a symphony of pre-mRNA processing activities. Biochim Biophys Acta 1577: 308–324. 12213660 10.1016/s0167-4781(02)00460-8

[27] Goldstrohm AC, Albrecht TR, Sune C, Bedford MT, Garcia-Blanco MA (2001) The transcription elongation factor CA150 interacts with RNA polymerase II and the pre-mRNA splicing factor SF1. Mol Cell Biol 21: 7617–7628. 11604498 10.1128/MCB.21.22.7617-7628.2001 PMC99933

[28] Edmunds JW, Mahadevan LC, Clayton AL (2008) Dynamic histone H3 methylation during gene induction: HYPB/Setd2 mediates all H3K36 trimethylation. EMBO J 27: 406–420. 18157086 10.1038/sj.emboj.7601967 PMC2168397

[29] Nogues G, Kadener S, Cramer P, Bentley D, Kornblihtt AR (2002) Transcriptional activators differ in their abilities to control alternative splicing. Journal of Biological Chemistry 277: 43110–43114. 12221105 10.1074/jbc.M208418200

[30] Batsche E, Yaniv M, Muchardt C (2006) The human SWI/SNF subunit Brm is a regulator of alternative splicing. Nature Structural & Molecular Biology 13: 22–29. 10.1038/nsmb1030 16341228

[31] Luco RF, Pan Q, Tominaga K, Blencowe BJ, Pereira-Smith OM, et al (2010) Regulation of alternative splicing by histone modifications. Science 327: 996–1000. 20133523 10.1126/science.1184208 PMC2913848

[32] Barski A, Cuddapah S, Cui K, Roh TY, Schones DE, et al (2007) High-resolution profiling of histone methylations in the human genome. Cell 129: 823–837. 17512414 10.1016/j.cell.2007.05.009

[33] Wang Z, Zang C, Rosenfeld JA, Schones DE, Barski A, et al (2008) Combinatorial patterns of histone acetylations and methylations in the human genome. Nat Genet 40: 897–903. 18552846 10.1038/ng.154 PMC2769248

[34] Zhao KJ, Chepelev I, Wei G, Tang QS (2009) Detection of single nucleotide variations in expressed exons of the human genome using RNA-Seq. Nucleic Acids Research 37. 10.1093/nar/gkp507 PMC2760790 19528076

[35] Cui K, Zang C, Roh TY, Schones DE, Childs RW, et al (2009) Chromatin signatures in multipotent human hematopoietic stem cells indicate the fate of bivalent genes during differentiation. Cell Stem Cell 4: 80–93. 19128795 10.1016/j.stem.2008.11.011 PMC2785912

[36] Edgar R, Domrachev M, Lash AE (2002) Gene Expression Omnibus: NCBI gene expression and hybridization array data repository. Nucleic Acids Research 30: 207–210. 11752295 10.1093/nar/30.1.207 PMC99122

[37] Bernstein BE, Stamatoyannopoulos JA, Costello JF, Ren B, Milosavljevic A, et al (2010) The NIH Roadmap Epigenomics Mapping Consortium. Nat Biotechnol 28: 1045–1048. 20944595 10.1038/nbt1010-1045 PMC3607281

[38] Benjamini Y, Yekutieli D (2001) The control of the false discovery rate in multiple testing under dependency. Annals of Statistics 29: 1165–1188.

[39] Neugebauer KM (2002) On the importance of being co-transcriptional. Journal of Cell Science 115: 3865–3871. 12244124 10.1242/jcs.00073

[40] Kornblihtt AR (2007) Coupling transcription and alternative splicing. Adv Exp Med Biol 623: 175–189. 18380347 10.1007/978-0-387-77374-2_11

[41] Pandit S, Wang D, Fu XD (2008) Functional integration of transcriptional and RNA processing machineries. Curr Opin Cell Biol 20: 260–265. 18436438 10.1016/j.ceb.2008.03.001 PMC2701685

[42] Nilsen TW, Graveley BR (2010) Expansion of the eukaryotic proteome by alternative splicing. Nature 463: 457–463. 20110989 10.1038/nature08909 PMC3443858

[43] Schwartz S, Meshorer E, Ast G (2009) Chromatin organization marks exon-intron structure. Nature Structural & Molecular Biology 16: 990–995. 10.1038/nsmb.1659 19684600

[44] Andersson R, Enroth S, Rada-Iglesias A, Wadelius C, Komorowski J (2009) Nucleosomes are well positioned in exons and carry characteristic histone modifications. Genome Research 19: 1732–1741. 19687145 10.1101/gr.092353.109 PMC2765275

[45] Han JDJ, Yu H, Zhu SS, Zhou B, Xue HL (2008) Inferring causal relationships among different histone modifications and gene expression. Genome Research 18: 1314–1324. 18562678 10.1101/gr.073080.107 PMC2493438

[46] van Steensel B, Braunschweig U, Filion GJ, Chen M, van Bemmel JG, et al (2010) Bayesian network analysis of targeting interactions in chromatin. Genome Research 20: 190–200. 20007327 10.1101/gr.098822.109 PMC2813475

[47] Zhang C, Frias MA, Mele A, Ruggiu M, Eom T, et al (2010) Integrative modeling defines the Nova splicing-regulatory network and its combinatorial controls. Science 329: 439–443. 20558669 10.1126/science.1191150 PMC3412410

[48] Gardina PJ, Clark TA, Shimada B, Staples MK, Yang Q, et al (2006) Alternative splicing and differential gene expression in colon cancer detected by a whole genome exon array. BMC Genomics 7: 325. 17192196 10.1186/1471-2164-7-325 PMC1769375

[49] Kwan T, Benovoy D, Dias C, Gurd S, Serre D, et al (2007) Heritability of alternative splicing in the human genome. Genome Research 17: 1210–1218. 17671095 10.1101/gr.6281007 PMC1933514

[50] Yeo GW, Xu X, Liang TY, Muotri AR, Carson CT, et al (2007) Alternative splicing events identified in human embryonic stem cells and neural progenitors. PLoS Comput Biol 3: 1951–1967. 17967047 10.1371/journal.pcbi.0030196 PMC2041973

[51] Magwene PM, Kim J (2004) Estimating genomic coexpression networks using first-order conditional independence. Genome Biol 5: R100. 15575966 10.1186/gb-2004-5-12-r100 PMC545795

[52] Jansen R, Greenbaum D, Gerstein M (2002) Relating whole-genome expression data with protein-protein interactions. Genome Res 12: 37–46. 11779829 10.1101/gr.205602 PMC155252

[53] Reverter A, Chan EK (2008) Combining partial correlation and an information theory approach to the reversed engineering of gene co-expression networks. Bioinformatics 24: 2491–2497. 18784117 10.1093/bioinformatics/btn482

[54] Chen L, Zheng SK (2009) Studying alternative splicing regulatory networks through partial correlation analysis. Genome Biology 10. 10.1186/gb-2009-10-1-r3 PMC2687791 19133160

[55] Butte AJ, Kohane IS (2000) Mutual information relevance networks: functional genomic clustering using pairwise entropy measurements. Pac Symp Biocomput: 418–429. 10.1142/9789814447331_0040 10902190

[56] Carter SL, Brechbuhler CM, Griffin M, Bond AT (2004) Gene co-expression network topology provides a framework for molecular characterization of cellular state. Bioinformatics 20: 2242–2250. 15130938 10.1093/bioinformatics/bth234

[57] Efron B (2007) Correlation and large-scale simultaneous significance testing. Journal of the American Statistical Association 102: 93–103.

[58] Zhang B, Horvath S (2005) A general framework for weighted gene co-expression network analysis. Stat Appl Genet Mol Biol 4: Article17. 16646834 10.2202/1544-6115.1128

[59] Celniker SE, Dillon LA, Gerstein MB, Gunsalus KC, Henikoff S, et al (2009) Unlocking the secrets of the genome. Nature 459: 927–930. 19536255 10.1038/459927a PMC2843545

[60] Bannister AJ, Schneider R, Myers FA, Thorne AW, Crane-Robinson C, et al (2005) Spatial distribution of di- and tri-methyl lysine 36 of histone H3 at active genes. J Biol Chem 280: 17732–17736. 15760899 10.1074/jbc.M500796200

[61] Talasz H, Lindner HH, Sarg B, Helliger W (2005) Histone H4-lysine 20 monomethylation is increased in promoter and coding regions of active genes and correlates with hyperacetylation. J Biol Chem 280: 38814–38822. 16166085 10.1074/jbc.M505563200

[62] Boyer LA, Plath K, Zeitlinger J, Brambrink T, Medeiros LA, et al (2006) Polycomb complexes repress developmental regulators in murine embryonic stem cells. Nature 441: 349–353. 16625203 10.1038/nature04733

[63] Kim S, Kim H, Fong N, Erickson B, Bentley DL (2011) Pre-mRNA splicing is a determinant of histone H3K36 methylation. Proceedings of the National Academy of Sciences of the United States of America 108: 13564–13569. 21807997 10.1073/pnas.1109475108 PMC3158196

[64] Enroth S, Bornelov S, Wadelius C, Komorowski J (2012) Combinations of histone modifications mark exon inclusion levels. PLoS One 7: e29911. 22242188 10.1371/journal.pone.0029911 PMC3252363

[65] Lee JS, Smith E, Shilatifard A (2010) The language of histone crosstalk. Cell 142: 682–685. 20813257 10.1016/j.cell.2010.08.011 PMC3711869

[66] Zippo A, Serafini R, Rocchigiani M, Pennacchini S, Krepelova A, et al (2009) Histone crosstalk between H3S10ph and H4K16ac generates a histone code that mediates transcription elongation. Cell 138: 1122–1136. 19766566 10.1016/j.cell.2009.07.031

[67] Wang Z, Zang C, Cui K, Schones DE, Barski A, et al (2009) Genome-wide mapping of HATs and HDACs reveals distinct functions in active and inactive genes. Cell 138: 1019–1031. 19698979 10.1016/j.cell.2009.06.049 PMC2750862

[68] Zhang P, Du J, Sun B, Dong X, Xu G, et al (2006) Structure of human MRG15 chromo domain and its binding to Lys36-methylated histone H3. Nucleic Acids Res 34: 6621–6628. 17135209 10.1093/nar/gkl989 PMC1747190

[69] Zeng L, Zhou MM (2002) Bromodomain: an acetyl-lysine binding domain. FEBS Lett 513: 124–128. 11911891 10.1016/s0014-5793(01)03309-9

[70] Pray-Grant MG, Daniel JA, Schieltz D, Yates JR, 3rd, Grant PA (2005) Chd1 chromodomain links histone H3 methylation with SAGA- and SLIK-dependent acetylation. Nature 433: 434–438. 15647753 10.1038/nature03242

[71] Schreiber SL, Bernstein BE (2002) Signaling network model of chromatin. Cell 111: 771–778. 12526804 10.1016/s0092-8674(02)01196-0

[72] Kent WJ, Sugnet CW, Furey TS, Roskin KM, Pringle TH, et al (2002) The human genome browser at UCSC. Genome Research 12: 996–1006. 12045153 10.1101/gr.229102 PMC186604

[73] Gabriel K, Sokal R (1969) A new statistical approach to geographic variation analysis. Systemat Zool 18: 20.

