# Supplementary figures and images for: Modeling Exon Expression Using Histone Modifications
Source: PLoS One. 2013 Jun 25;8(6):e67448. doi: 10.1371/journal.pone.0067448 (PMC3692485; doi:10.1371/journal.pone.0067448)

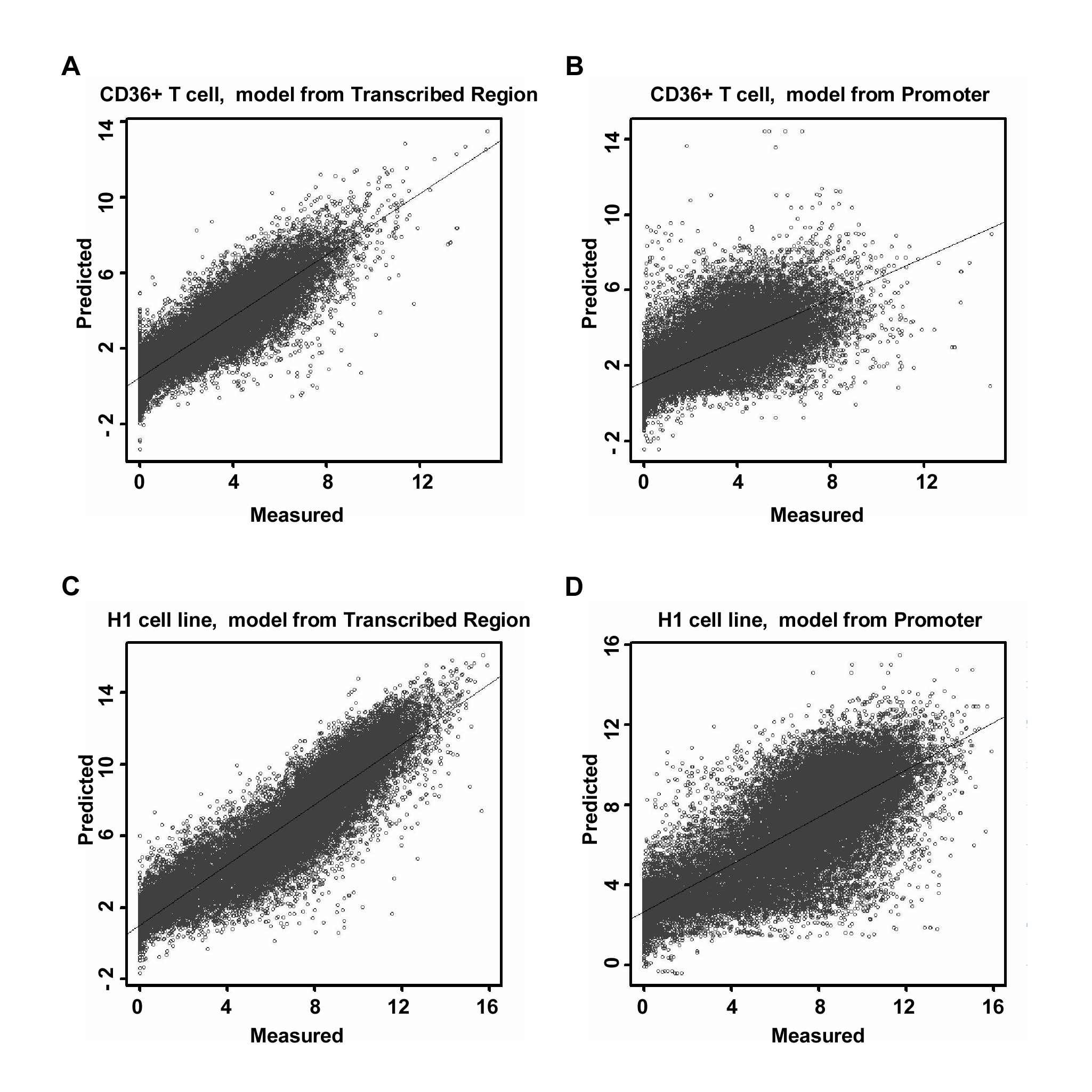

Supplement: Figure S1 — Histone modifications along transcribed regions predict gene expression more faithfully than those on promoters. The x axis represents the measured value of gene expression. The y axis represents the predicted value by the linear regression model using the histone modification levels as input. (A-B) The scatterplots with predicted and measured gene expression values for transcribed regions and promoters in the CD36+ T cell. (C-D) The scatterplots with predicted and measured gene expression values for transcribed regions and promoters in the H1 cell line. (TIF) [file pone.0067448.s001.tif]

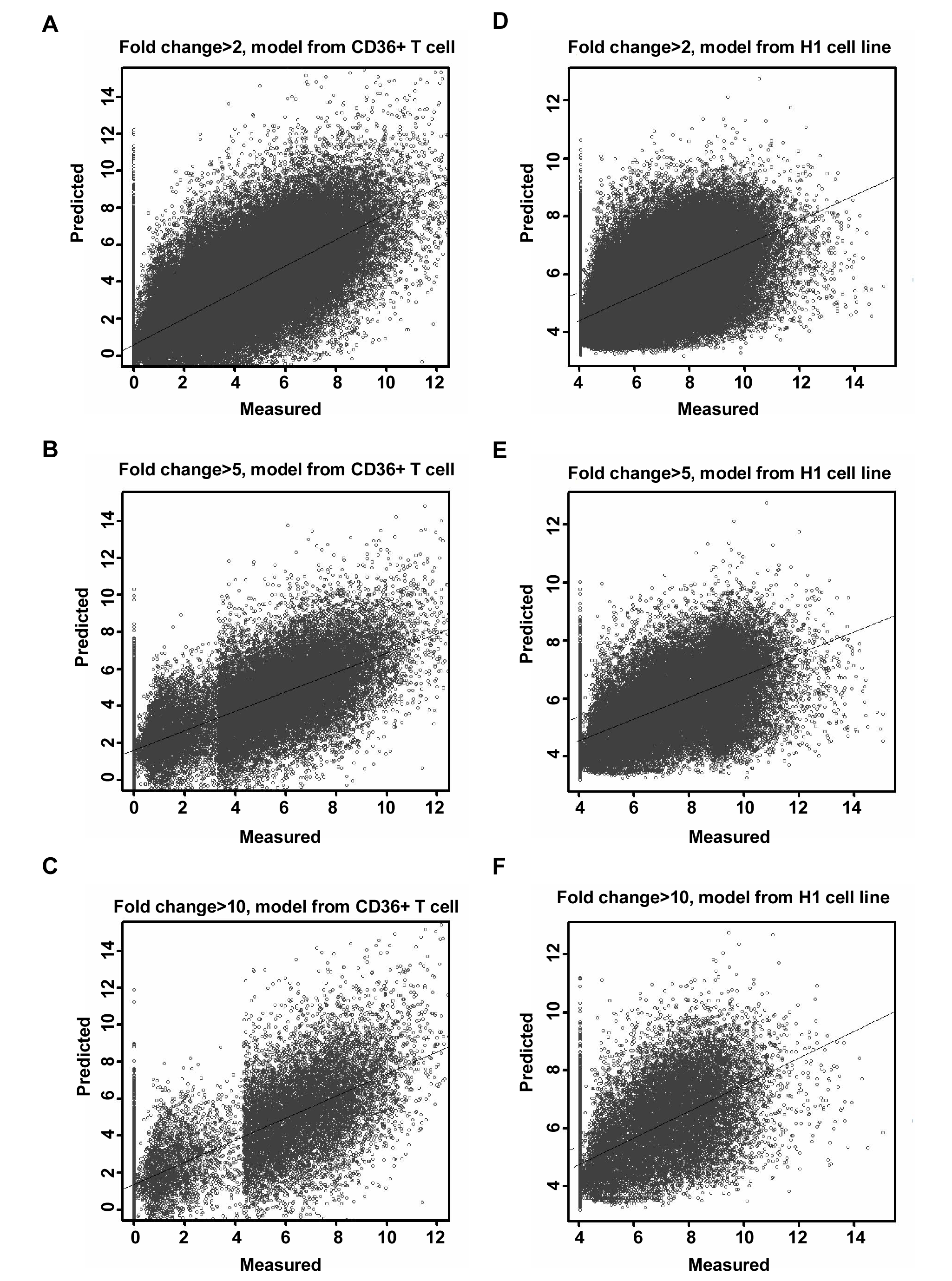

Supplement: Figure S2 — Prediction of exon expression across cell types. The x axis represents the measured value of exon expression. The y axis represents the predicted value by linear regression model using histone modification levels as input. (A-C) The prediction of exon expression in the CD4+ T cell using the linear regression model built from the CD36+ T cell. The linear regression model was trained on exons in the CD36+ T cell. Based on the resulting model, the histone modification levels on exons of the CD4+ T cell were employed as input to predict the corresponding exon expression values. This analysis was performed on exons whose expression changed at least 2-fold (A), 5-fold (B) and 10-fold (C) between two cell types. (D-F) The prediction of exon expression in the CD4+ T cell using the linear regression model built from the H1 cell line. The linear regression model was trained on exons in the H1 cell line. Based on the resulting model, the histone modification levels on exons of the CD4+ T cell were employed as input to predict the corresponding exon expression values. This analysis was performed on exons whose expression changed at least 2-fold (D), 5-fold (E) and 10-fold (F) between two cell types. (TIF) [file pone.0067448.s002.tif]

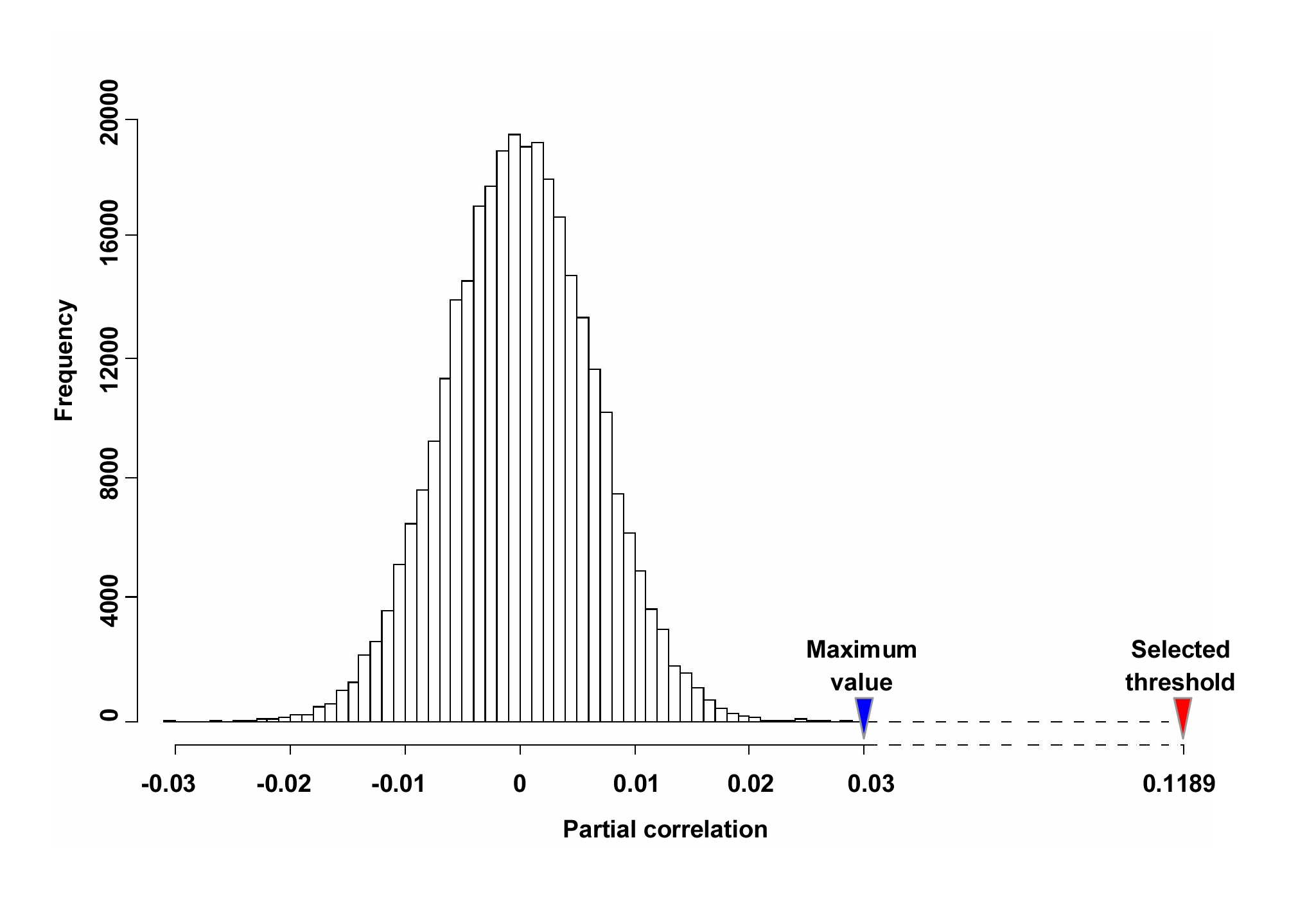

Supplement: Figure S3 — The significance of the selected threshold was validated using the permutation test. The histogram for the pair-wise partial correlations of the permutation. It illustrates the frequencies of partial correlation coefficients for the permutation. The blue and red triangles respectively represent the maximum partial correlation coefficient generated by permutation and the selected threshold for the interaction network. (TIF) [file pone.0067448.s003.tif]

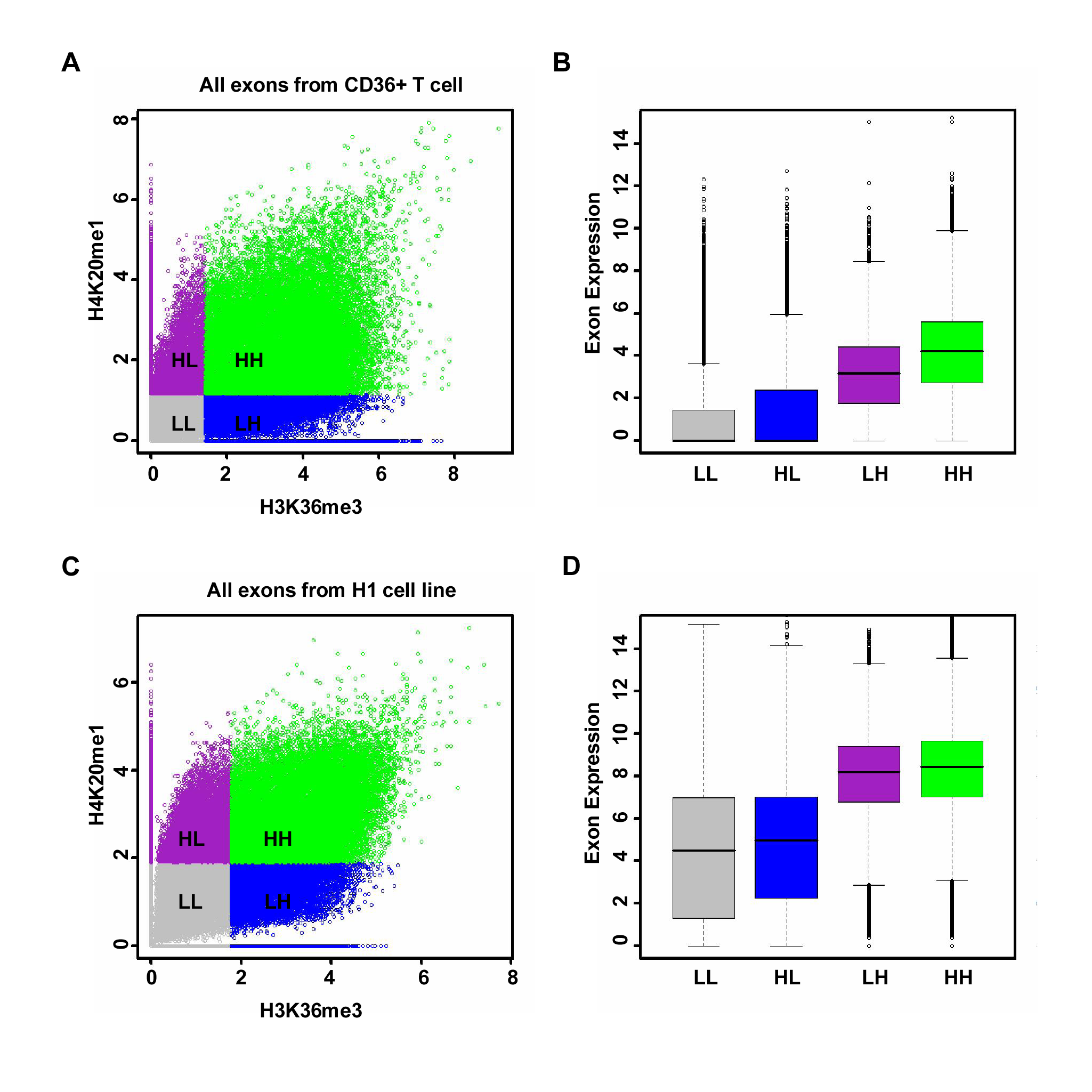

Supplement: Figure S4 — Co-regulation of histone modification combinations in the CD36+ T cell and H1 cell line. (A and C) The x and y axis respectively represent the intensities of corresponding histone modifications. In the CD36+ T cell and H1 cell line, all exons were respectively grouped into four bins: LL (grey), HL (purple), LH (blue) and HH (green). Whether the intensity is H or L is determined by comparing the histone modification intensity with the corresponding median value (1.43 for H3K36me3 and 1.16 for H4K20me1 in the CD36+ T cell; 1.77 for H3K36me3 and 1.91 for H4K20me1 in the H1 cell line). (B and D) The distributions of the exon expression for the four bins in two cell types. (TIF) [file pone.0067448.s004.tif]

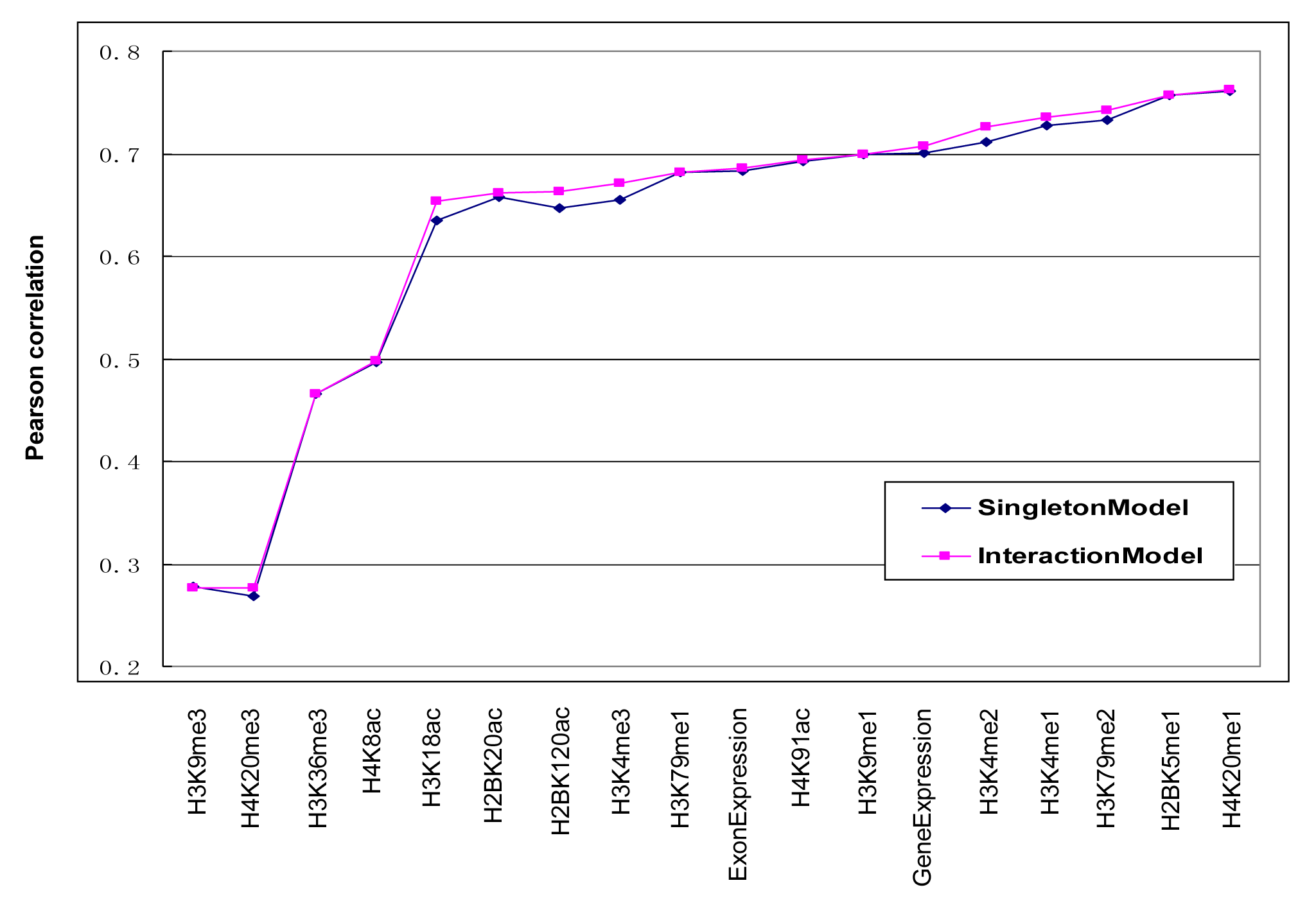

Supplement: Figure S5 — The interaction model did not lead to obvious improvement compared to the singleton model. Singleton and interaction regression models were respectively constructed according to eighteen histone modification combinations indicated by the partial correlation network. The x axis denotes the explanatory variables in eighteen regression models. The y axis represents Pearson correlation coefficients between measured and predicted values. The explanatory variables were sorted by ascending Pearson correlation coefficients along the x axis. (TIF) [file pone.0067448.s005.tif]
